# Genomic analysis of Shiga toxin-producing *Escherichia coli* O157:H7 from cattle and pork-production related environments

**DOI:** 10.1038/s41538-021-00097-0

**Published:** 2021-07-01

**Authors:** Peipei Zhang, Saida Essendoubi, Julia Keenliside, Tim Reuter, Kim Stanford, Robin King, Patricia Lu, Xianqin Yang

**Affiliations:** 1 Agriculture and Agri-Food Canada, Lacombe, Alberta Canada; 2 Alberta Agriculture and Forestry, Edmonton, Alberta Canada; 3 Alberta Agriculture and Forestry, Lethbridge, Alberta Canada; 4 University of Lethbridge, Lethbridge, Alberta Canada

**Keywords:** Bacteria, Microbial ecology

## Abstract

Three *E. coli* O157:H7 outbreaks have been attributed to contaminated pork in Alberta, Canada, recently. This study investigates the phylogenetic relatedness of *E. coli* O157:H7 from pigs, cattle, and pork-production environments for source attribution. Limited strain diversity was observed using five conventional subtyping methods, with most or all strains being in one subgroup. Whole-genome single nucleotide polymorphism analysis confirmed the recent ancestry of the isolates from all three sources. Most environmental isolates clustered closer with pig isolates than cattle isolates. Also, a direct link was observed between 2018-outbreak environmental isolates and isolates collected from a pig farm in 2018. The majority of pig isolates harbor only one Shiga toxin gene, *stx*_*2a*_, while 70% (35/50) of the cattle isolates have both *stx*_*1a*_ and *stx*_*2a*_. The results show some *E. coli* O157:H7 strains could establish persistence on pig farms and as such, pigs can be a significant source of the organism.

## Introduction

Shiga toxin-producing *Escherichia coli* (STEC) can cause human enteric illness ranging from uncomplicated diarrhea to bloody diarrhea, with a fraction (2–22%) of patients developing life-threatening hemolytic uremic syndrome (HUS)^1–6^. The predominant STEC serotype associated with human infections and outbreaks in North America is O157:H7/NM, which also has a higher association with severe patient outcomes, compared to other serotypes^1,7^.

Beef has been frequently implicated in STEC O157 outbreaks^1,7^. Ruminants including cattle, are regarded as a natural reservoir of STEC O157:H7^8^. On the contrary, swine is not a recognized reservoir of STEC O157:H7, and their contribution to human STEC O157 infections has been a matter for debate^9^. Unlike cattle which are asymptomatic carriers, pigs can develop edema when infected with STEC strains harboring *stx*_*2e*_^10^. Outbreaks attributed to STEC O157:H7 contaminated pork products are scarce worldwide^11^; however, a higher frequency has been observed in Alberta, Canada, with three outbreaks having occurred in four years (2014, 2016, and 2018)^12–14^. The outbreak in 2014 was the second-largest foodborne outbreak in Canadian history with 119 lab-confirmed cases, while the 2018 outbreak had 42 confirmed cases with one death likely due to the infection with *E. coli* O157:H7^12,13^.

A large number of studies from various geographical regions have been conducted to investigate the prevalence of STEC O157:H7 in healthy pigs^15–33^. Most of these studies did not find this pathogen in pigs^16–20,22,25–27,31,33^, except for the studies from Japan^32^, Ireland^23^, UK^24^, Sweden^28^, Norway^30^, USA^29^, and Canada^15^ where up to 2% prevalence was reported. Among these studies, a recent survey performed at 39 provincially inspected abattoirs in Alberta found 1.4% of pigs and 1.8% of pork carcasses were positive for STEC O157:H7, and 12.8% of abattoirs had at least one positive sample^15^. In addition, an outbreak-related investigation in Alberta in 2018 recovered STEC O157:H7 from pig fecal samples on the outbreak-associated farms (Keenliside, unpublished data). It is not clear whether pigs are transient carriers of STEC O157, or some strains of the pathogen can circulate on pig farms, with pigs being the primary reservoir.

With the decrease in cost and increase in throughput for WGS, subtyping methods for surveillance and phylogenetic analysis of pathogenic bacteria have been rapidly evolving from analyzing a limited number of sites/genes in a bacterial genome to genome-wide comparisons. Analysis based on single nucleotide polymorphism (SNP) and core genome (cg)/whole genome (wg) multilocus sequence typing (MLST) are the most often used WGS subtyping methods, followed by gene content analysis^34–37^. Despite the much improved discriminatory power of WGS-based methods^38,39^, it is still of value to link findings from WGS-based analysis to conventional subtyping methods, and make use of the large body of information in the literature. Thus, in the present study, we analyzed the population structure and phylogenetic relatedness of 121 STEC O157:H7 isolates from pigs (*n* = 41), cattle (*n* = 51), and pork-production environments (*n* = 30) in Alberta, Canada using both conventional and WGS-based subtyping methods. Also, the genomes of these isolates were characterized with a focus on virulence and antibiotic resistance genes.

## Results

### Subtyping of *E. coli* O157:H7 isolates using conventional methods

All 121 *E. coli* O157:H7 isolates (Table S1) were subtyped for Manning clade^40^, lineage-specific polymorhpism (LSPA)^41^, MLST type, and Clermont phylo-group^42^ via *in silico* analysis. Most of the isolates (115/121, 95%) were identified to be of Manning clade 2 although cattle isolates showed more diversity (Table 1). The clades 4-7 and 9 strains share the same SNP profile for the target genes and hence cannot be differentiated by the clade typing method. Of the 121 isolates, 115 belonged to LSPA lineage I. Again, cattle isolates showed more diversity, with Cat02-Cat04 and Cat13 belonging to lineage I/II, and Cat46 belonging to lineage II. Cat24 lacked the *rbsB* gene and as such, could not be subtyped by LSPA^41^. Using the Pasteur Institute’s eight-gene scheme, Env06 (from a pig carcass) and Cat12 did not match any MLST type in the database as the two isolates had a unique allele for the target genes *polB* and *icdA*, respectively. Cat02-Cat04, Cat13 and Cat46 were identified as MLST type 628 and all the other isolates as 822. Neither Achtman’s seven-gene MLST scheme nor the Clermont’s *E. coli* phylo-typing method^42^ was able to distinguish the isolates, with all being identified as MLST type 11 or phylo-group E, respectively (Table 1).

**Table 1:** The conventional subtyping of 121 *E. coli* O157:H7 isolates from various sources.

Isolates	Manning clade	Lineage	MLST type		Clermont phylo-group
			Pasteur Institute’s eight-gene scheme	Achtman’s seven-gene scheme	
All pig isolates	2	I	822	11	E
Env01-Env05, Env07-Env30	2	I	822	11	E
Env06	2	I	Uncertain^c^	11	E
Cat01, Cat05-Cat11, Cat14-Cat19, Cat21-Cat23, Cat25-Cat45, Cat47-Cat50	2	I	822	11	E
Cat02-Cat04, Cat13	4/5/6/7/9^a^	I/II	628	11	E
Cat12	2	I	Uncertain^c^	11	E
Cat20	3	I	822	11	E
Cat24	2	Uncertain^b^	822	11	E
Cat46	4/5/6/7/9^a^	II	628	11	E
O157:H7 EDL933 (Reference)	3	I	822	11	E
O157:H7 Sakai (Reference)	1	I	296	11	E

^a^The isolates belonged to one of clades 4, 5, 6, 7, and 9. The subtyping scheme described by Riordan et al. (2008) cannot differentiate strains of clade 4, 5, 6, 7, and 9.

^b^The amplicon for the marker gene, *rbsB* (Yang et al., 2004) was not predictable using *in silico* PCR and the draft genome of Cat24 and hence no lineage was assigned for this isolate.

^c^The isolates did not match any MLST type in Pasteur Institute’s eight-gene scheme although the closest MLST type of both isolates was 822.

### Comparison of WGS-based subtyping methods

**Fig. 1:**
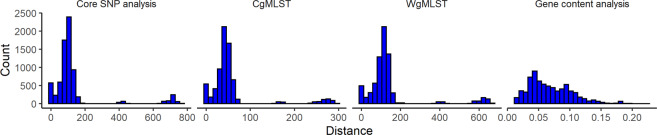
Distribution of genetic distance for *E. coli* O157:H7 (*n* = 121) isolated from pigs, cattle, and pork production environment. The distance was calculated from core SNP analysis (SNPs), cgMLST (allelic distance), wgMLST (allelic distance), and gene content analysis (Jaccard distance). Y-axis represents the number of combinations. There were a total number of 7260 combinations for the 121 genomes.

The phylogenetic relationships among the isolates were further mined using core SNP analysis, cgMLST, wgMLST, and gene content analysis. The core SNPs between the reference strain *E. coli* O157:H7 EDL 933^43^ and the 121 isolates included 2565 sites in total. CgMLST and wgMLST included 2513 and 24952 genes, respectively. The distances among isolates were 0–770 SNPs, 0–297 alleles, 0–669 alleles, and 0.012–0.222 (Jaccard distance) for core SNP analysis, cgMLST, wgMLST, and gene content analysis, respectively (Fig. 1; Tables S2–S5).

**Fig. 2:**
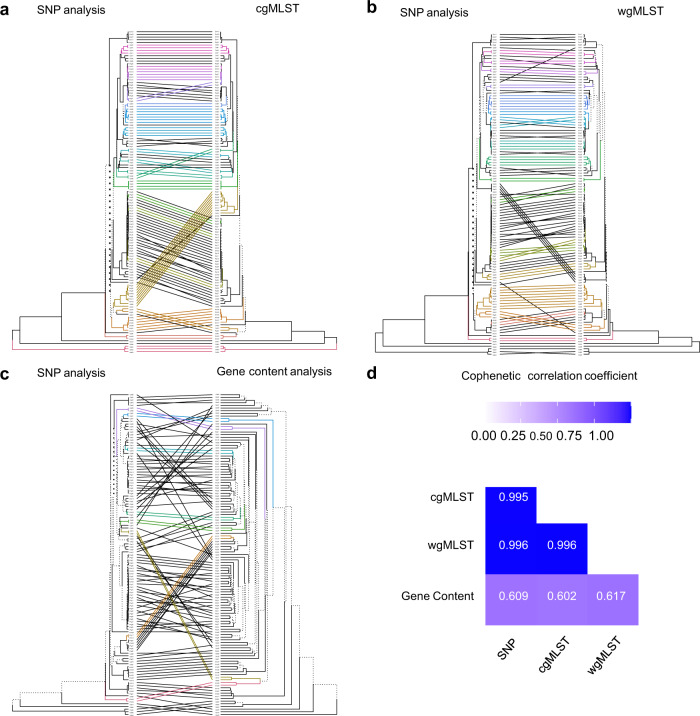
Comparison of UPGMA dendrograms from four WGS-based subtyping methods. **a**–**c** shows the comparison between dendrograms produced from core SNP analysis and cgMLST (**a**), wgMLST (**b**), and gene content analysis (**c**), respectively. **d** shows the pairwise Cophenetic correlation coefficients of dendrograms. In **a**–**c**, the same tips/isolates of two trees are connected using straight lines; the connecting lines and branches are colored to highlight the sub-trees that are present in both dendrograms; the nodes which contain a combination of labels/items that are only present in one of the two trees are highlighted using dashed lines.

**Fig. 3:**
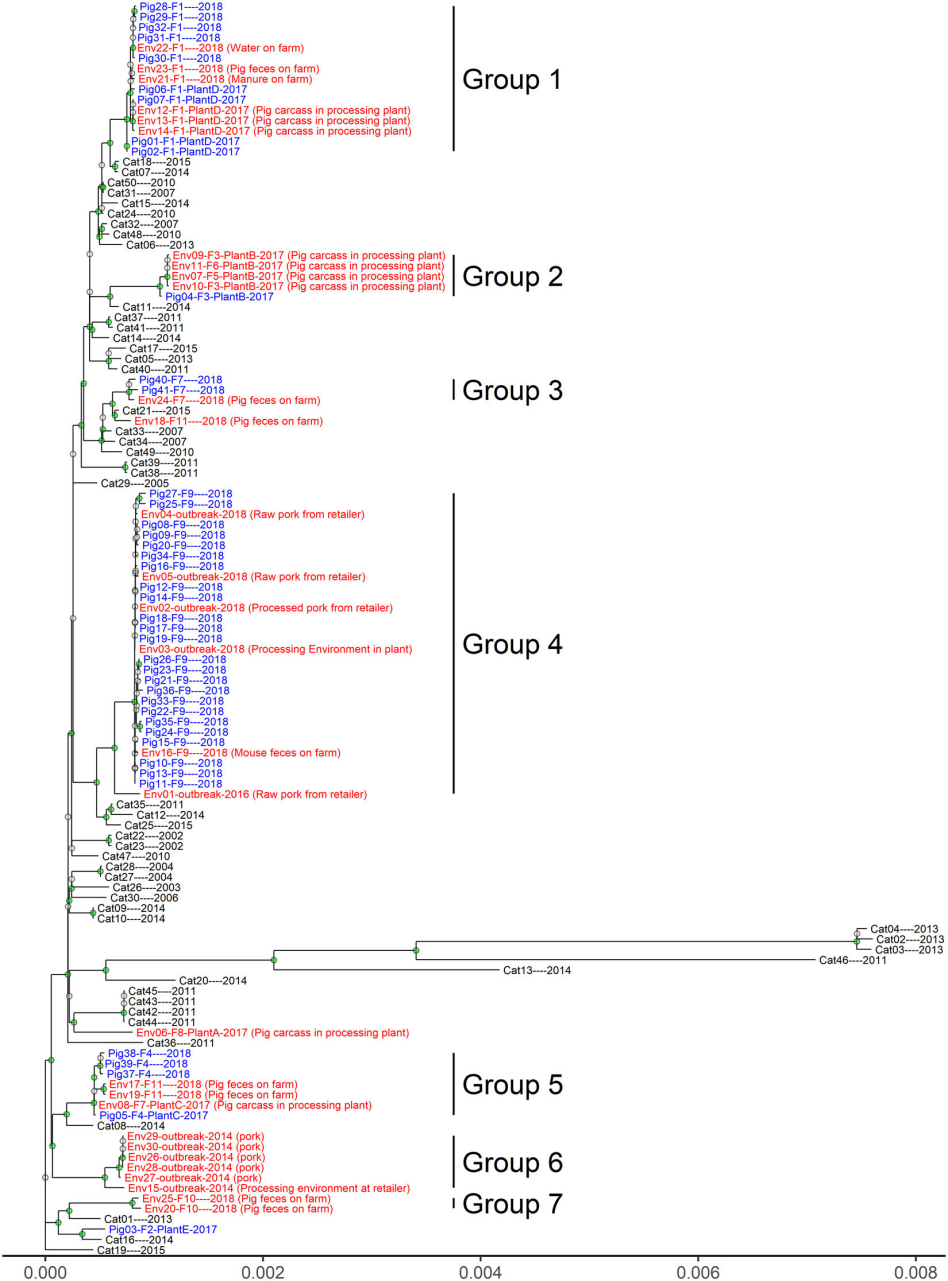
A maximum likelihood tree based on core SNPs of EDL933 (Reference) and 121 *E. coli* O157:H7 isolates from pigs, cattle and pork production environment. The nodes with bootstrap support value >70 are labeled green. The substitution(s) per site is represented in the scale bar. Each tip in the tree was labeled with the isolate’s identification and, if applicable, its associated farm (F), and/or processing plant (Plant) followed by the year of collection. The pig isolates (blue) and cattle (black) isolates were recovered from fresh fecal/cecal samples of both animal species. The environment isolates (red) were recovered from various samples (information added in the parenthesis following the tree tips) collected from the pork production and distribution chain. Porcine and environmental isolates primarily clustered in seven groups in the tree, as labeled in the figure.

**Fig. 4:**
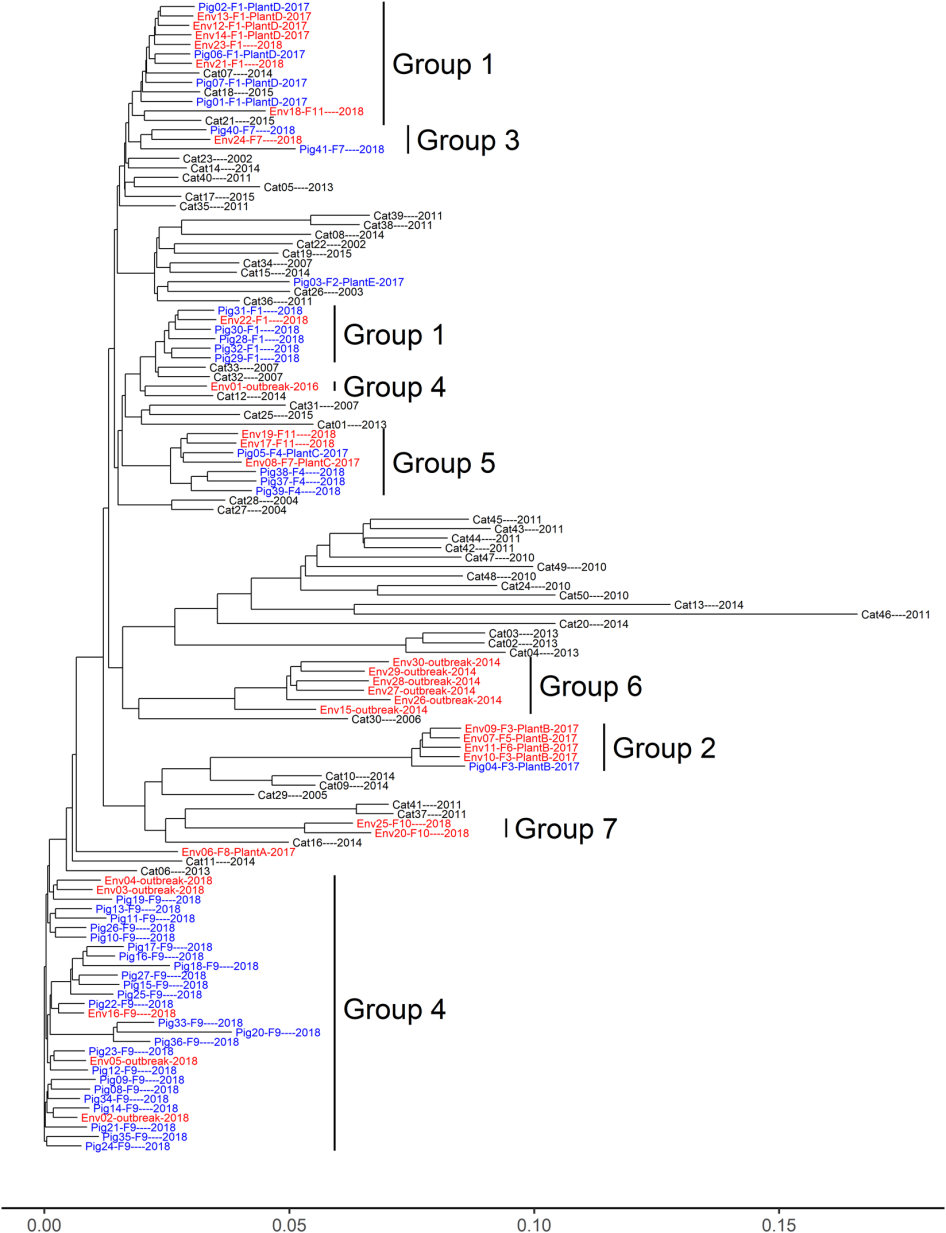
A neighbor joining tree based on the gene content of 121 *E. coli* O157:H7 isolates from pigs, cattle and pork production environment. The scale bar represents the Jaccard distance based on the presence or absence of genes between genomes. Each tip in the tree was labeled with the isolate’s identification and, if applicable, its associated farm (F), and/or processing plant (Plant) followed by the year of collection. The isolates recovered from pig (blue) and cattle (black) fecal samples and pig production environment samples (red) were distinguished using different colors. The branches of the seven groups formed by pig and environment isolates in core SNP tree (Fig. 3) are labeled in text in the figure. The groups 2, 3, 5, 6, and 7 were also clustered together in this tree, however, groups 1 and 4 were separated into two respectively subgroups.

Distance-based dendrograms constructed using the unweighted pair group method with arithmetic mean (UPGMA) showed significant correlation/similarity (*P* < 0.01) among the four dendrograms (Fig. 2). The pair-wise Cophenetic correlation coefficient (CPCC)^44^ among dendrograms from core SNP analysis, cgMLST, and wgMLST was between 0.995 and 0.996 (Fig. 2). The CPCC between any of the three dendrograms and that from gene content analysis was between 0.602 and 0.617, suggesting the gene content dendrogram was only moderately correlated with the former three dendrograms. Consistent with the statistical analyses, visual comparisons showed that the phylogenetic trees from core SNP analysis, cgMLST and wgMLST clustered the isolates in a similar structure/topology while the gene content tree differed from the rest (Fig. 3 & 4 and S1-S2). For example, the core SNP analysis, cgMLST and wgMLST trees clustered pig and pig production environment isolates mainly into seven groups (Fig. 3, S1 and S2). In the gene content tree (Fig. 4), groups 2, 3, 5, 6, and 7 pig and pig environment isolates were in their respective distinct clusters, while groups 1 and 4 isolates were each separated into two subgroups, suggesting differences in gene gain and/or loss among the isolates within each of the two groups.

### Phylogenetic relatedness among the *E. coli* O157 isolates

**Fig. 5:**
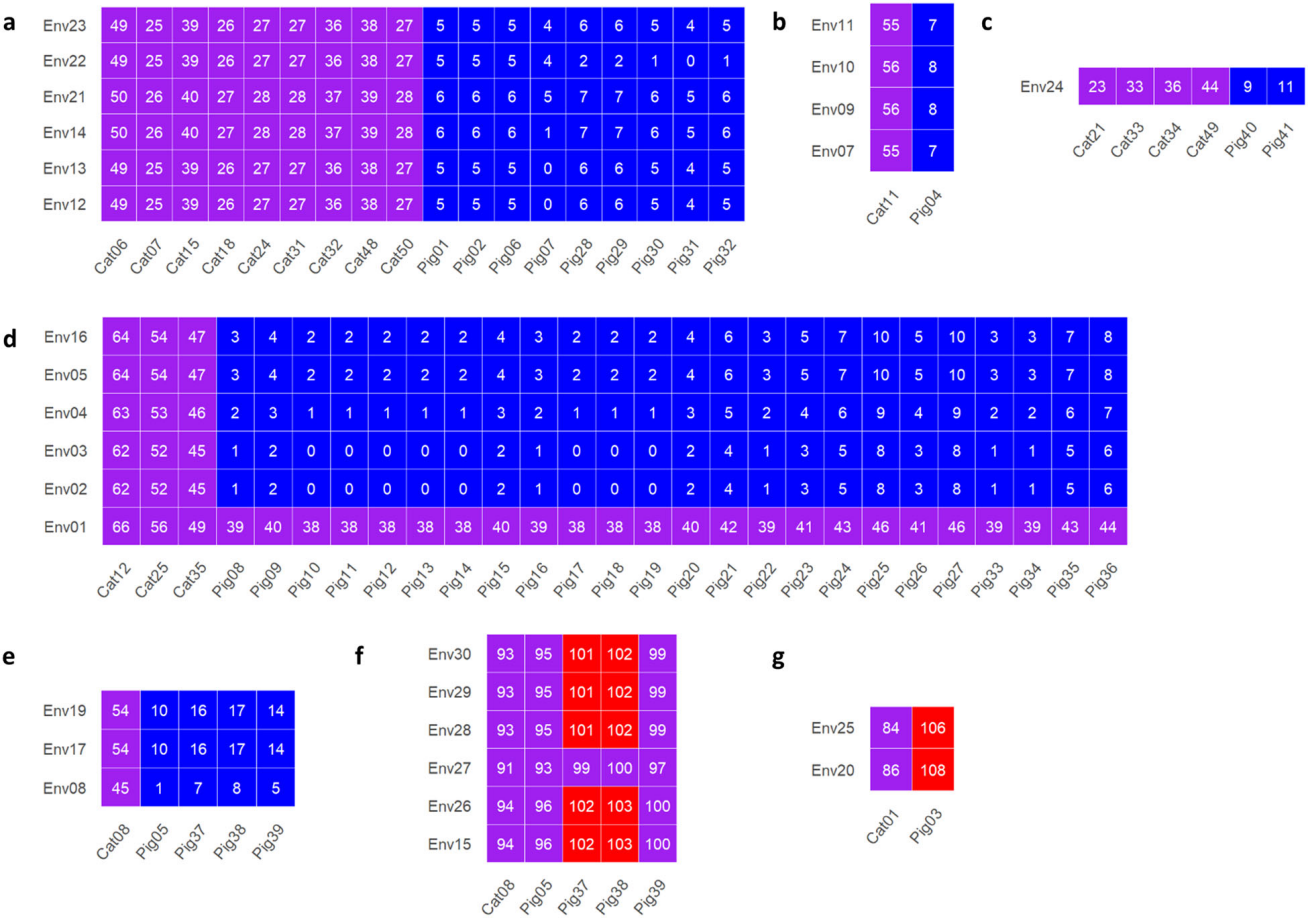
The SNP distance of environmental isolates of each phylogenetic group with their closest cattle and pig isolates. **a**–**g** shows the environmental isolates of group 1–7, respectively. The SNP distances are coded with blue, purple, and red representing <21 SNPs, ≥21 SNPs and ≤100 SNP, and >100 SNPs, respectively.

The core SNP analysis was selected for interpretation of the phylogenetic relationship among the bacterial isolates. As mentioned above, environmental and/or pig isolates mostly clustered together, forming seven phylogenetic groups, 1–7, except for a few isolates (Env18, an isolate from pig feces on farm 11; Env06, from pig carcasses in plant A; Pig03, from pigs from farm 2) which clustered together with one or more cattle isolate(s) individually (Fig. 3; Table S2). Cut-off values of ≤20, >20 and ≤100 and >100 SNPs have been recommended to cluster bacterial isolates in food surveillance studies as closely related, possibly related and not related, respectively^45^. Based on this definition, most of the isolates within each of groups 1–7 were closely related (Table S2), except for Env01 (2016-outbreak isolate) which had genetic distances of 38–46 SNPs in comparison to all other isolates in group 4. Group 1–5 environmental isolates clustered closer with pig isolates than cattle isolates (Fig. 5a–e). The distance between environmental isolates of group 1–5 and their respective closest pig isolate(s) was 0–7, 7–8, 9–11, 0–46 and 1–17 SNPs, respectively. In contrast, the distance between group 1 and 5 environmental isolates and their respective closest cattle isolate(s) was ≥25, 55, 23, 45, and 45 SNPs. Groups 6–7 environmental isolates had distances of ≥84 SNPs with either pig or cattle isolates included in this study (Fig. 5f, g). All porcine and environmental isolates had genetic distance of ≤100 SNPs with at least one of the cattle isolates (Fig. S3), suggesting a recent divergence in ancestry of these isolates.

**Fig. 6:**
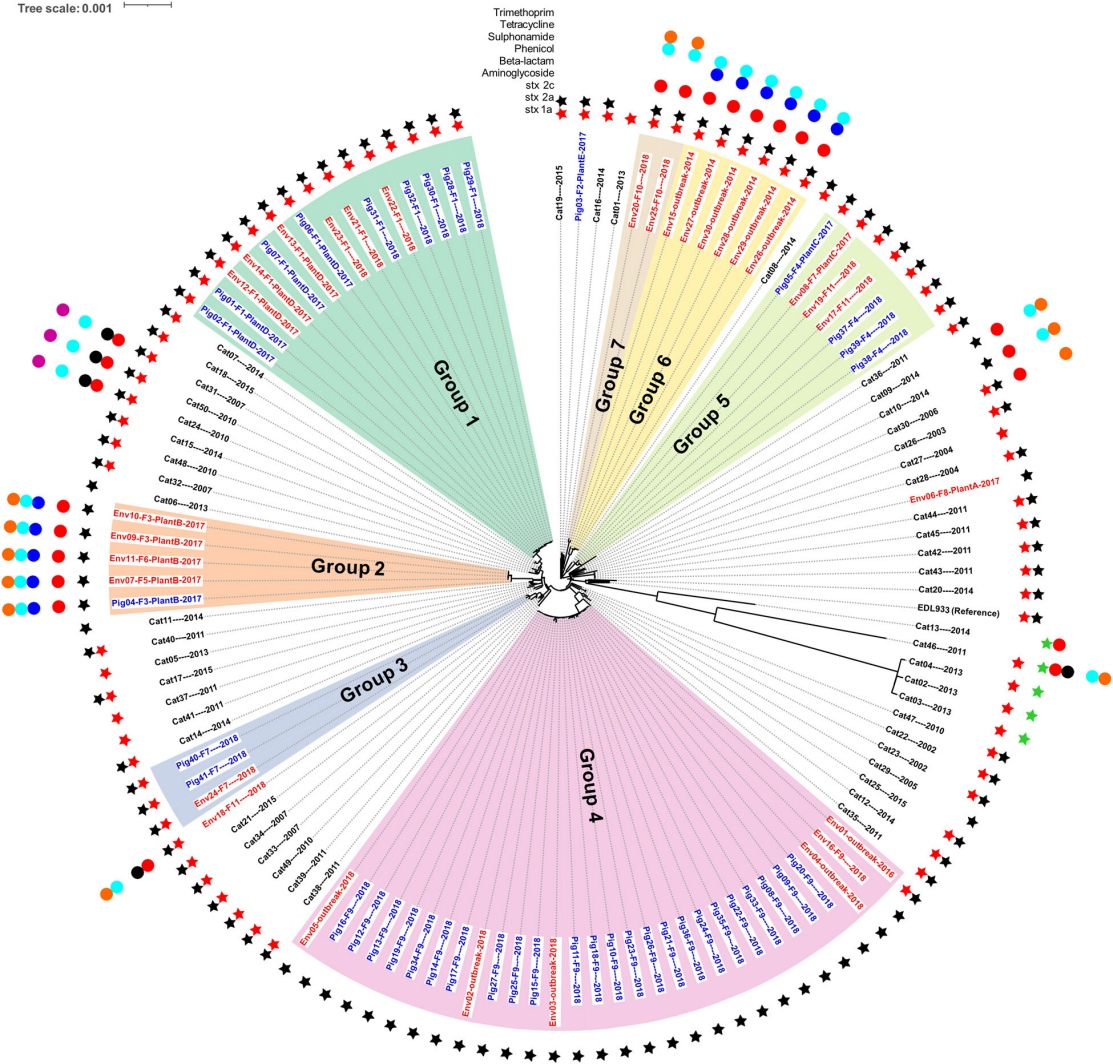
The core SNP tree shown in the circular. The tip text for cattle, pig, and pig production environment isolates is print in black, blue, and red, respectively. The format is “isolate id—year of collection” for cattle isolates and “isolate id –associated farm (if available) –associated plant (if available)-year of collection” for pig and environment isolates, respectively. The seven phylogenetic groups formed by pig and environment isolates are highlighted with different colors and labeled with text. The presence of Shiga toxin-producing genes *stx*_*1a*_, *stx*_*2a*_, and *stx*_*2c*_ is shown as a red, black, and green star, respectively. The resistance to classes of antibiotics for each isolate, including aminoglycoside, beta-lactam, phenicol, sulfonamide, tetracycline, and trimethoprim, is shown as a red, black, dark blue, light blue, orange, and purple circle, respectively.

The pig and environmental isolates were recovered from pig fecal samples or environmental samples collected from 11 farms (1–11) or four meat processing plants (a–d) (Figs. 3 and 6). Closely related pig isolates were recovered from farms 1 and 4 over a period of two years and frequently from pigs on farm 9 (Figs. 3 and 6). The genetic distances between the 2018-outbreak isolates (Env02-Env05, Fig. 5d) and pig isolates from farm 9 were ≤10 SNPs, indicating a strong association between this outbreak and farm 9 pigs. Both the 2014-outbreak isolates (Env15, Env26-Env30; Fig. 5f) and a 2016-outbreak isolate (Env01, Fig. 5d) had similar genetic distance to their respectively closest pig (≥93 SNPs, 2014; ≥38 SNPs, 2016) and cattle (≥91 SNPs, 2014; ≥49 SNPs, 2016) isolates.

### Pan-genome

In total, 8096 genes were found in the pan-genome of the 121 isolates, including 4052 (50.05%) core genes (shared by all 121 isolates) and 4044 (49.95%) accessory genes (present in between 1 and 120 isolates) (Fig. S4). The database for Clusters of Orthologous Groups of proteins (COGs)^46^ was used to classify the functional categories of each gene in the pan-genome. In total, 745 core and 2871 accessory genes were not classified. Of note, among the classified genes in the accessory genome, category X (mobilome: prophages, transposons) accounted for the largest proportion (38.9%), indicating the large contribution of this category to the plasticity of *E. coli* O157:H7 genomes.

### Virulence genes

Genes encoding Shiga toxin (*stx*) subtypes 1a, 2a and 2c were found in the 121 STEC O157:H7 genomes, with the patterns of “1a only”, “2a only”, “2c only”, “1a and 2a”, “1a and 2c” (Fig. 6; Table 2). Of the 41 pig isolates, 61% (25) harbored *stx*_*2a*_ only and all the other pig isolates (16; 39%) had both *stx*_*1a*_ and *stx*_*2a*_, while 33.3% (10) and 66.7% (20) of environmental isolates had *stx*_*2a*_ only and *stx*_*1a*_ and *stx*_*2a*_, respectively (Table 2). The most abundant pattern among cattle isolates was “1a and 2a” (35, 70%), followed by “1a only” (5, 10%), “2a only” (5, 10%), “1a and 2c” (4, 8%) and “2c only” (1, 2%). The *stx* profiles correlated with their respective phylogenetic groups (Fig. 6). For example, group 1, 3, 5, and 6 isolates harbored both *stx*_1a_ and *stx*_*2a*_, while group 2 and group 4 (except isolate Env01 from the 2016-outbreak) had *stx*_*2a*_ only.

**Table 2:** The number (percentage, %) of *E. coli* O157:H7 isolates with different Shiga toxin-producing gene profile.

	Pig isolates	Cattle isolates	Environmental isolates
*Stx*_*1a*_ only	0 (0)	5 (10)	0 (0)
*Stx*_*2a*_ only	25 (61)	5 (10)	10 (33.3)
*Stx*_*2c*_ only	0 (0)	1 (2)	0 (0)
*Stx*_*1a*_ *+ Stx*_*2a*_	16 (39)	35 (70)	20 (66.7)
*Stx*_*1a*_ *+ Stx*_*2c*_	0 (0)	4 (8)	0 (0)
Total	41 (100)	50 (100)	30 (100)

Besides *stx*, 319 other virulence genes were found in the genomes (Table S6). The gene *eae* located on the locus of enterocyte effacement (LEE), encoding intimin, was found in all but Cat48 (Table S6). The gene *paa* encoding the porcine attaching/effacing-associated protein^47^ and *toxB* encoding an adhesion protein^48^ were absent in only one genome, Cat13. *EhaA* encoding an autotransporter protein that contributes to adhesion and biofilm formation^49^ was found in all genomes. The genes *saa* and *lpfA* which encode an autoagglutinating adhesion or long polar fimbriae in LEE negative STEC^50,51^, respectively, were not found in any of the genomes. Three genes encoding potential adhesion proteins *c3610*, *cah*, *iha*^52^ were absent in all phylogenetic group 2 isolates, but present in most other isolates. α-hemolysin encoding gene *ehxA*^53^ was found in all genomes but Cat45. A heat-stable enterotoxin (EAST1) encoding gene *astA*^54^ was found in all isolates. Another thermostable enterotoxin gene *estIA* often found in enteroaggregative *E. coli*^55^ or a subtilase cytotoxin encoding gene *subA*^56^ was not found in any of the genomes.

### Antibiotic resistance

Of the 121 *E. coli* O157:H7 isolates, 22 (18.2%) were predicted to be resistant to at least one of six classes of antibiotics including aminoglycoside, beta-lactam, phenicol, sulfonamide, tetracycline, and/or trimethoprim (Fig. 6). The 2014-outbreak isolates (group 6) were resistant to aminoglycosides, phenicols, and sulfonamides while the 2016- and 2018-outbreak isolates were predicted not to be resistant to any group of the antibiotics. Group 2 and 7 isolates were resistant to four and three antibiotic classes, respectively. On the contrary, group 1, 3, 4, and 5 isolates, which were associated with farm 1/plant D, farm 7, farm 9, and farm 4/farm 11/plant C, respectively, were predicted not to be resistant to any of the antibiotics examined.

### Plasmid sequences

Bacterial plasmids often carry virulence and antibiotic resistance genes^57^. The program PlasmidFinder^58^ evaluates a replicon sequence as a signature to identify plasmid sequence-bearing contigs. In total, 11 variants of replicons were found in the genomes, including Col(BS512), Col156, IncFIA, IncFIB(AP001918), IncFII, IncFII(pHN7A8), IncFII(pCoo), IncI2(Delta), IncI1-I(Gamma), IncN, and pEC4115 (Table S7).

All isolates had replicon sequences of IncFIB (AP001918) and IncFII. Isolates Cat02-04 and Cat46 all had IncFIA as well. A Blast screening against the NCBI nucleotide database found that the contigs containing these replicon sequences all had a >99% identity with the megaplasmid, pO157 often present in *E. coli* O157:H7 genomes^59^. The pO157 marker gene *ehxA* and relevant transport protein-encoding genes *etpC-O* were found in these contigs.

A larger percentage of cattle isolates (26%, 13/50) carried other plasmids/replicons compared to pig isolates (2/41, 4.9%). For pig and environmental isolates, the presence of replicons was related to their phylogenetic group with groups 2, 6, and 7 having Col(BS512) and IncI1-I(Gamma), IncFII(pCoo), IncFII(pCoo) and IncI1-I(Gamma), respectively (Table S7). The IncN replicon in Env18 (recovered from pig feces on farm 11) had resistance genes to four classes of antibiotics including *aph(3”)-Ib* and *aph(6)-Id*, *blaTEM-1B*, *sul2,* and *tet(A)* for aminoglycosides, beta-lactams, sulfonamides, and tetracyclines, respectively. The plasmids with IncFII(pCoo) in group 6 isolates harbored genes encoding resistance to aminoglycosides (*aadA2b* and *aadA1*), phenicols (*cmlA1*) and sulfonamides (*sul3*). For isolates Cat50, Cat31, and Cat24, the plasmids with IncFII(pHN7A8) carried the resistance genes for aminoglycosides (*aph(3”)-Ib* and *aph(6)-Id*) and sulfonamides (*sul2*). However, in the genome of Cat30 the IncFII(pHN7A8)-containing contig only carried aminoglycoside resistance genes *(aph(3”)-Ib* and *aph(6)-Id)*.

## Discussion

This study investigated the phylogenetic relatedness of STEC O157:H7 (*n* = 121) recovered from pigs, cattle, and pork-production-related environments in Alberta, Canada, using both conventional subtyping schemes and WGS-based methods. To date, a paucity of information on the genetic relationship between cattle and swine STEC strains is available in published accounts. *E. coli* O157:H7 strains from the two animal species showed distinct randomly amplified polymorphic DNA (RAPD) patterns in a Korean study^60^, while similar pulsed-field gel electrophoresis (PFGE) patterns were found in a Norwegian study^30^. The present study observed very limited strain diversity for isolates from both animal species determined by all five conventional subtyping methods: 95%, 95%, 94.2%, 100%, and 100% of the isolates belonging to one Manning clade (2), LPSA lineage (I), MLST type (822; Pasteur Institute’s eight-gene scheme), MLST type (11, Achtman’s seven-gene scheme) or Clermont phylo-group (E). Genetic variation of *E. coli* O157 can be driven by geographical locations^61–64^. Published data reviewed by Pianciola and Rivas^63^ showed that the most predominant Manning clade of *E. coli* O157:H7 in the USA, Argentina, and Australia is 2, 8, and 7, respectively. Strachan et al.^64^ showed the predominant *E. coli* O157:H7 in Canada between 1995 and 2012 is clade 2, followed by clade 3. LSPA lineage I have been reported to be predominant among cattle *E. coli* O157:H7 isolates in Canada, followed by lineage II and I/II^63^, which is consistent with the findings in this study.

The four WGS-based subtyping methods all showed a much higher discriminatory power compared to conventional typing methods, as would be expected. Phylogenetic trees from the former three subtyping methods congruously separated the porcine and environmental isolates mainly into seven phylogenetic groups, with cgMLST having the lowest resolution. However, the gene content analysis further separated group 1 and group 4 into two subgroups, with different positioning for some isolates, and divided clonal isolates, especially in the phylogenetic trees based on SNP and cgMLST analysis into well-defined branches. SNP analysis uses the genome of a closely related bacterial strain as a reference and infers phylogeny among bacterial strains based on the variants in both intragenic and intergenic regions shared by both reference and query genomes^34^. On the other hand, cgMLST and wgMLST analyze the allelic difference of genes in the core (cgMLST) and pan (wgMLST) genomes, respectively^34^. For both cgMLST and wgMLST, the missing alleles are ignored in pairwise comparison and one allelic difference is counted for a given gene if two genomes have different alleles no matter how many SNPs are found in this gene. The difference in discriminatory power of SNP and cg/wgMLST was then to be expected. A gene content-based method infers the phylogeny based on the presence or absence of genes in the pan-genome of included bacterial strains and hence reflects difference in the accessory genome rather than the core genome^36,37^. The differences between the accessory genome tree (gene content analysis) and core genome trees (SNP and cgMLST) are in agreement with the previous observations on the plasticity of *E. coli* genomes, with horizontal gene transfer being the primary driving force for the evolution of *E. coli*^37,65^. The observation that some cattle isolates (e.g. Cat07 and Cat18) intermingled with pig isolates suggest that those pig isolates may have originated from cattle or a common source with cattle isolates, and that a subgroup of cattle isolates may have acquired accessory genomes to adapt to the pig gut environment.

Pightling et al.^45^ suggested a cutoff value of 21 and 100 SNPs for defining clonal strains and possibly related strains of *E. coli* for outbreak investigations. All porcine and environmental isolates had genetic distance of <100 SNPs with at least one cattle isolate included in this study, suggesting the recent common ancestry shared between *E. coli* O157:H7 from both animal species. This is consistent with the low strain diversity observed by conventional subtyping methods. The genetic differences among isolates within each phylogenetic group of pig and environmental isolates are mostly less than 20 SNPs. A large number of clonal STEC O157:H7 strains, as determined by SNP analysis, were frequently recovered from pigs (farm 9, ≤13 SNPs; farm 1, ≤7 SNPs). The prevalence of STEC O157:H7 in pigs is low in most geographical areas globally and whether swine are competent carriers of this pathogen has long been debated^9^. A study carried out by Wöchtl and co-workers^66^ reported clinical symptoms including diarrhea, dehydration, and neurological disorders in piglets infected with STEC O157:H7. In contrast, Booher et al.^67^ found pigs can be persistently colonized by STEC O157:H7 for >2 months. The repeated recovery of the clonal strain of STEC O157 from pigs on individual farms over an extended period of time in the present study supports the latter of the two studies. The 2018-outbreak isolates and pig isolates from farm 9 differed by less than 10 SNPs. To the authors’ best knowledge, a direct link between STEC O157:H7 of pig origin and an outbreak found in this study was not reported before. The 2016-outbreak isolates and farm 9 isolates recovered in 2018 may share a recent common ancestor as they differed only by 38–46 SNPs. In addition, most (66.7%, 20/30) environmental isolates are more closely related to pig isolates than cattle isolates. These findings suggest that pigs can be a significant source of STEC O157 contamination on pork and in pork-production environments.

Shiga toxins, carried by prophages, are the defining feature of STEC. There are two types of Shiga toxins, Stx1 and Stx2, each further divided into several subtypes (Stx1a and Stx1c-1e; Stx2a-2i, Stx2k, and Stx2l) based on the serological reaction and the amino acid sequence^1^. Stx2e can cause edema disease in pigs^68^. In general, Stx2 is more associated with the human disease than Stx1, with the subtype Stx_2a_ being often associated with patients with HUS than other subtypes^1,69–71^. A study of Chui et al.^72^ reported negative interactions between Stx1a and Stx2a, proposing that STEC O157 isolates carrying only Stx2a are more virulent than those with both Stx1 and Stx2a. The cattle and pig/environmental isolates had different *stx* gene profiles. The cattle isolates mostly carried *stx*_*1a*_ and *stx*_*2a*_, and this agrees with previous reports on the *stx* profile of STEC O157 recovered from Alberta cattle^73^. On the other hand, most pig isolates carried *stx*_*2a*_ only. An Indonesian study reported that STEC O157:H7 from pigs (*n* = 7) all had *stx*_*2a*_^74^; however, only *stx*_*2a*_ was examined in that study. There are a total of 14 genomes of STEC O157:H7 pig isolates deposited in GenBank (2 from China and 12 from USA), with six, six, and two of the isolates having both *stx*_*1a*_ and *stx*_*2a*_, *stx*_*2c*_ and *stx*_*1a*_, respectively (Table S8). Studies reporting the recovery of STEC O157:H7 from pigs are very limited. A number of studies on non-O157 STEC from pigs found *stx*_*2e*_ to be the predominant *stx* subtype^9,19,75,76^. The *eae* gene was present in all of the isolates included in this study except for Cat48, which is in agreement with the prevalence of this gene in STEC O157 in published accounts^73,77–83^. All the isolates have the gene *ehxA*, located on the megaplasmid pO157^59^. In addition, most of the STEC O157:H7 isolates also harbor additional genes involved in intestinal adhesion (e.g. *paa, toxB,* and *ehaA*), encoding other toxins (e.g. *astA*), involved in type II/III secretion system, and encoding fimbrial proteins. The distribution of these virulence genes does not seem to be associated with origin of isolation, i.e., pigs, cattle, or environment.

Only one (Pig04) of the 41 pig STEC O157 isolates was predicted to be antibiotic-resistant. On the contrary, a relatively larger proportion of cattle isolates, 8/50, harbored antibiotic resistance genes. Resistance to at least two antibiotic classes have been found for 16% of O157 isolates from Alberta feedlots cattle^84^. Among non-type specific *E. coli* recovered from cattle in Alberta feedlots, the resistance to tetracyclines, aminoglycosides (represented by streptomycin), and sulfonamides (represented by sulfisoxazole) have been the most commonly detected^85^. The pattern of antibiotic resistance in environmental isolates was closely related to the phylogenetic group, with group 2, 6, and 7 isolates and Env18 (from pig feces on farm 11) predicted to be resistant to at least one type of antibiotics. This suggests potential cross-contamination between two animal species as plant B processed both pork and beef and farm 10 had both cattle and swine during the period in which the isolates were recovered.

Unlike the plasmid pO157, other plasmids carried by *E. coli* O157:H7 are not well studied. Rusconi et al.^38^ investigated the plasmids in STEC O157:H7 associated with seven outbreaks in the USA and found plasmids of the incompatible groups IncI1, IncI2, IncK, IncFII, and pEC4115. The preliminary analysis in this study found the plasmids with replicon sequences Col (BS512), Col156, IncFII (pHN7A8), IncFII (pCoo), IncI2 (Delta), IncI1-I(Gamma), IncN and pEC4115 in STEC O157:H7 isolates, with IncN, IncFII (pCoo) and IncFII (pHN7A8) plasmids carrying genes resistant to four, three and two/one classes of antibiotics including aminoglycosides, beta-lactams, sulfonamides, tetracyclines, and phenicols. Similar to the present pattern of antibiotic resistance genes, a larger proportion of cattle isolates carried additional plasmid(s).

In conclusion, pig and pork-production environmental STEC O157:H7 isolates were classified mainly into seven distinct yet related phylogenetic groups, with each group primarily associated with the specific farm(s)/plant(s). The results demonstrated that some STEC O157:H7 strains originating from pigs can establish persistence on farms, and that pigs can be a significant source for STEC O157:H7 to contaminate pork, and of the environmental dissemination. Different antibiotic resistance and *stx* profiles were observed for STEC O157:H7 isolates originating from Alberta cattle and pigs, with strains from the latter having a higher proportion of the more virulent *stx*_*2a*_ subtype.

## Methods

### Bacterial isolates

A total of 121 STEC O157:H7 isolates from pigs (*n* = 41; Pig01-41), pork-production environments (*n* = 30; Env01-30), and cattle (*n* = 50; Cat01-50) in Alberta, Canada, were included in this study^15,86–94^ (Stanford et al., unpublished data; Reuter et al., unpublished data; Keenliside et al., unpublished data) (Table S1). Pig and cattle isolates were recovered from fecal samples of pigs and cattle, respectively. The environmental isolates were recovered from various types of samples collected from the pork production and distribution environments including retailers, pork processing facilities, and pig barns/lagoons. Among the environmental isolates, Env15 and Env26-Env30 (2014 outbreak), Env01 (2016), and Env02-Env05 (2018) were associated with the three *E. coli* O157:H7 outbreaks attributed to contaminated pork in Alberta. Cattle isolates were recovered from the feces of cattle from various feedlots or at two slaughter plants in Alberta, Canada between 2002 and 2015. The pig and environmental isolates were from 11 farms (1–11) and four meat processing plants (A-D) between 2014 and 2018. We sequenced the genomes of Pig01-41, Cat01-12, Cat14-19, Cat21-23, Cat25-41, and Env01-25^95^. The genomes of Cat13, Cat20, Cat24, and Cat42-50^87,89^ were downloaded from the National Center for Biotechnology Information (NCBI) GenBank database. For Env26-30^93^, the raw sequencing data were downloaded from GenBank and assembled as described by Zhang, et al.^95^.

### Conventional genotyping using WGS data

To determine the Manning clade of the isolates, the SNPs in four target genes including ECs2357, ECs2521, ECs3881, and ECs4130 were analyzed^96^. The clade for each isolate was then assigned as described previously^96^. For LSPA lineage, a perl script (https://github.com/egonozer/in_silico_pcr) and the primers designed by Yang et al.^41^. were used to perform *in silico* PCR. For MLST, mlst (https://github.com/tseemann/mlst) was used, with both Achtman’s seven-gene scheme and Pasteur Institute’s eight-gene scheme. The Clermont *E. coli* phylo-typing^42^, which assigns phylo-group to *E. coli* based on the presence/absence of the genes *arpA*, *chuA*, and *yjaA* and an anonymous DNA fragment TspE4.C2, was also performed.

### Whole-genome SNP analysis and core SNP tree

For SNP analysis, Snippy 4.4.0 (https://github.com/tseemann/snippy) was used with the complete genome of EDL933 (ATCC 43895) as a reference (GenBank Accession no.: chromosome: CP008957.1, plasmid: CP008958.1)^43^. For all isolates except Cat13, Cat20, Cat24, and Cat42-Cat50, trimmed sequencing reads were used as input for “snippy” with default settings. The isolates Cat13, Cat20, Cat24, and Cat42-Cat50 only had genome sequences available, and as such, –contigs options were used for “snippy”. The contig-option shreds the contigs into synthetic reads and calls SNPs based on the generated reads. The recombination sites in the alignment file were removed using Gubbins^97^. Pairwise SNP distance matrix was generated using snp-dists (https://github.com/tseemann/snp-dists). A best-scoring maximum likelihood tree based on core SNP alignment was constructed using RAxML version 8 with general time reversible gamma nucleotide model (GTRGAMMA) and 100 times of bootstrap analyses^98^. iTOL v4^99^ and the ggtree package^100^ in R were both used to display and annotate the phylogenetic tree.

### cgMLST and wgMLST

ChewBBACA^101^ was used to perform both cgMLST and wgMLST. The published *Escherichia* schemes were downloaded from Enterobase (http://enterobase.warwick.ac.uk/schemes/). Both schemes were respectively adapted to chewBBACA and loci which did not meet the following criteria were removed: both valid start and stop codons were present in the coding sequence; the sequence length must be a multiple of 3; the sequence does not contain ambiguous character, i.e. containing ATGC only. The final schemes included 2513 and 24952 genes for cgMLST and wgMLST, respectively. The alleles were called with the default settings in chewBBACA. Neighbor joining trees were generated using GrapeTree^102^ based on the allele profiles of cgMLST and wgMLST, respectively, with the missing alleles ignored for pairwise comparison.

### Pan-genome analysis and gene content tree

The 121 genomes were annotated using Prokka v1.13.7^103^. The pan-genome of the isolates was parsed using Roary^104^, with the identity threshold to cluster proteins set at 90%. The functional category of the annotated coding sequences was assigned as described by Zhang et al.^105^. The Jaccard distances based on the presence or absence of genes between genomes were calculated using the dist() function in R with the method option set at “binary”^106^. A neighbor-joining tree was created using phangorn package^107^ in R.

### Comparison of WGS-based subtyping methods

To compare the four WGS subtyping methods, the distance matrices from four methods were used to generate UPGMA dendrograms via the hclust() function in R. For pairwise visual comparison, tanglegrams were created using the dendextend package in R. Cophenetic correlation coefficient was calculated for each pair of trees. To provide statistical support for the correlation, a permutation test was performed for each combination to produce one-sided *P* values. If *P* < 0.05, the similarity between two trees was regarded statistically significant.

### Virulence and antibiotic resistance genes

The presence of *stx* and their subtypes were determined using the webserver of the Center for Genomic Epidemiology (CGE) (https://cge.cbs.dtu.dk/services/VirulenceFinder/) with 90% as the threshold for identity and 80% for the minimum coverage, respectively^108^. For other virulence genes, the reference database ecoli_vf (June 15, 2020; https://github.com/phac-nml/ecoli_vf) and the virulence gene database (version, 2020-05-29) from CGE were both used. ABRicate v1.0.1 (https://github.com/tseemann/abricate) was used to scan the contig sequences of each genome against the reference databases. A match with >80% coverage and >90% identity was regarded as positive. Antibiotic resistance genes and plasmid sequences were identified using ResFinder^109^ and PlasmidFinder^58^ in CGE, respectively.

## Supplementary information

Supplementary Figures

Supplementary Tables S1-S8

## Data Availability

The authors declare that the data supporting the findings of this study are presented within the manuscript and supplementary files. The raw sequencing data and genome sequences can be found at: https://www.ncbi.nlm.nih.gov/bioproject/PRJNA661559.
