# Publisher Correction: Genomic analysis of Shiga toxin-producing *Escherichia coli* O157:H7 from cattle and pork-production related environments

**DOI:** 10.1038/s41538-021-00104-4

**Published:** 2021-07-19

**Authors:** Peipei Zhang, Saida Essendoubi, Julia Keenliside, Tim Reuter, Kim Stanford, Robin King, Patricia Lu, Xianqin Yang

**Affiliations:** 1 Agriculture and Agri-Food Canada, Lacombe, Alberta Canada; 2 Alberta Agriculture and Forestry, Edmonton, Alberta Canada; 3 Alberta Agriculture and Forestry, Lethbridge, Alberta Canada; 4 University of Lethbridge, Lethbridge, Alberta Canada

**Keywords:** Bacteria, Microbial ecology

Correction to: *npj Science of Food* 10.1038/s41538-021-00097-0, published online 1 July 2021

The copyright holder for this article was incorrectly given as ‘The Authors’, but should have been ‘Crown’. This has been corrected in the HTML and PDF version of the Article.

