# Supplementary material for: Genomic analysis of Shiga toxin-producing Escherichia coli O157:H7 from cattle and pork-production related environments
Source: NPJ Sci Food. 2021 Jul 1;5:15. doi: 10.1038/s41538-021-00097-0 (PMC8249597; doi:10.1038/s41538-021-00097-0)
Supplement: Supplementary file 1 — Supplementary Figures [file 41538_2021_97_MOESM1_ESM.pdf]

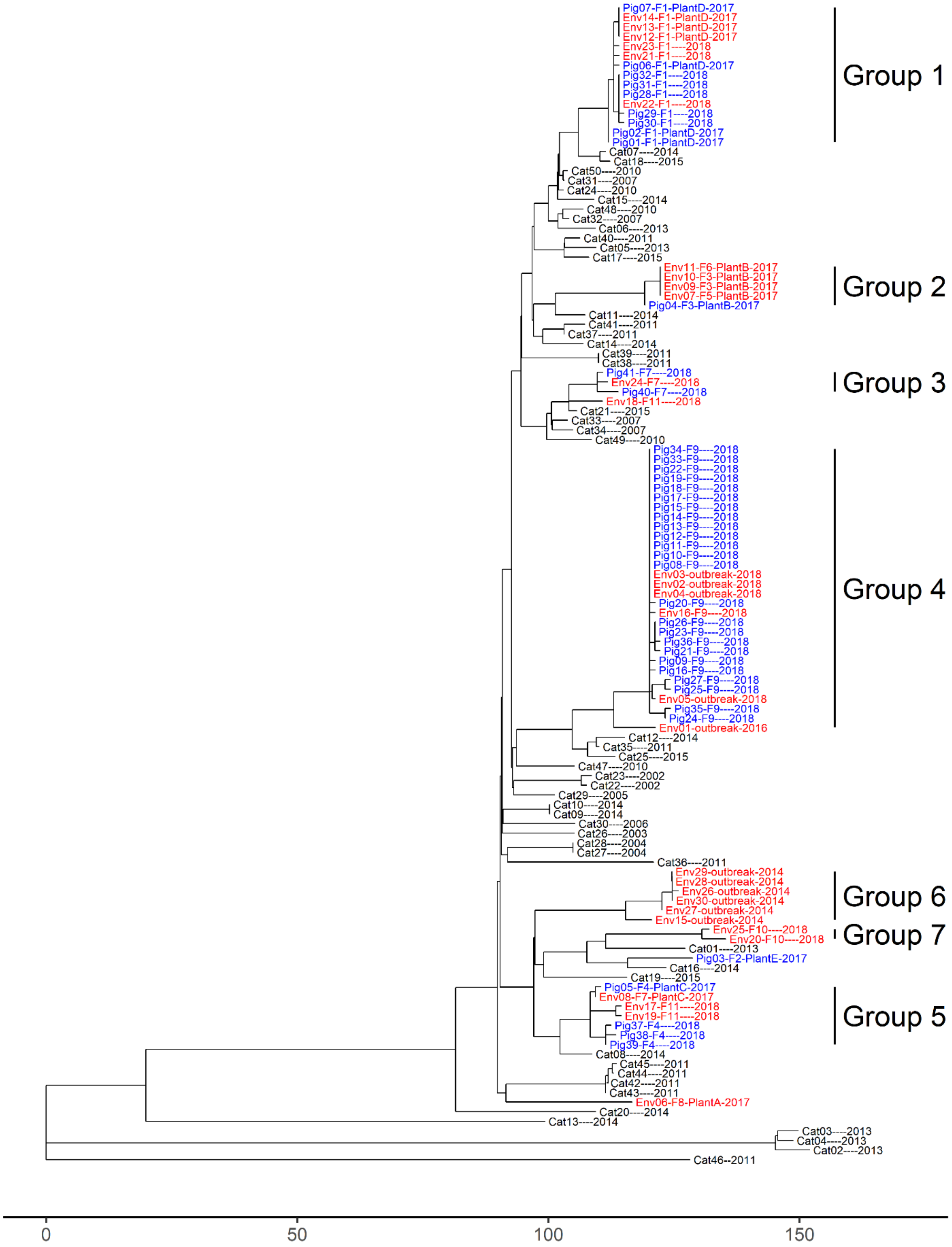
Pig07-F1-PlantD-2017
Env14-F1-PlantD-2017
Env13-F1-PlantD-2017
Env12-F1-PlantD-2017
Env23-F1----2018
Env21-F1----2018
Pig06-F1-PlantD-2017
Pig32-F1----2018
Pig31-F1----2018
Pig28-F1----2018
Env22-F1----2018
Pig29-F1----2018
Pig30-F1----2018
Pig02-F1-PlantD-2017
Pig01-F1-PlantD-2017
Cat07----2014
Cat18----2015
Cat50----2010
Cat31----2007
Cat24----2010
Cat15----2014
Cat48----2010
Cat32----2007
Cat06----2013
Cat40----2011
Cat05----2013
Cat17----2015
Env11-F6-PlantB-2017
Env10-F3-PlantB-2017
Env09-F3-PlantB-2017
Env07-F5-PlantB-2017
Pig04-F3-PlantB-2017
Cat11----2014
Cat41----2011
Cat37----2011
Cat14----2014
Cat39----2011
Cat38----2011
Pig41-F7----2018
Env24-F7----2018
Pig40-F7----2018
Env18-F11----2018
Cat21----2015
Cat33----2007
Cat34----2007
Cat49----2010
Pig34-F9----2018
Pig33-F9----2018
Pig22-F9----2018
Pig19-F9----2018
Pig18-F9----2018
Pig17-F9----2018
Pig15-F9----2018
Pig14-F9----2018
Pig13-F9----2018
Pig12-F9----2018
Pig11-F9----2018
Pig10-F9----2018
Pig08-F9----2018
Env03-outbreak-2018
Env02-outbreak-2018
Env04-outbreak-2018
Pig20-F9----2018
Env16-F9----2018
Pig26-F9----2018
Pig23-F9----2018
Pig36-F9----2018
Pig21-F9----2018
Pig09-F9----2018
Pig16-F9----2018
Pig27-F9----2018
Pig25-F9----2018
Env05-outbreak-2018
Pig35-F9----2018
Pig24-F9----2018
Env01-outbreak-2016
Cat12----2014
Cat35----2011
Cat25----2015
Cat47----2010
Cat23----2002
Cat22----2002
Cat29----2005
Cat10----2014
Cat09----2014
Cat30----2006
Cat26----2003
Cat28----2004
Cat27----2004
Cat36----2011
Env29-outbreak-2014
Env28-outbreak-2014
Env26-outbreak-2014
Env30-outbreak-2014
Env27-outbreak-2014
Env15-outbreak-2014
Env25-F10----2018
Env20-F10----2018
Cat01----2013
Pig03-F2-PlantE-2017
Cat16----2014
Cat19----2015
Pig05-F4-PlantC-2017
Env08-F7-PlantC-2017
Env17-F11----2018
Env19-F11----2018
Pig37-F4----2018
Pig38-F4----2018
Pig39-F4----2018
Cat08----2014
Cat45----2011
Cat44----2011
Cat42----2011
Cat43----2011
Env06-F8-PlantA-2017
Cat20----2014
Cat13----2014
Cat03----2013
Cat04----2013
Cat02----2013
Cat46--2011
Group 1
Group 2
Group 3
Group 4
Group 6
Group 7
Group 5
0
50
100
150

1

**Figure S1.** A neighbor joining tree based on cgMLST profiles of 121 *E. coli* O157:H7 isolates. The cgMLST scheme used included 2513 core genes. The pairwise allelic distances between allele profiles of 121 isolates were calculated and used to infer the tree. The scale bar represents the allelic distance. Each tip in the tree was labeled with the isolate identification and if applicable, its associated farm (F), and/or processing plant (Plant) followed by the year of collection. The isolates recovered from pig (blue) and cattle (black) fecal samples and pig production environment samples (red) were distinguished using different colors. The seven groups formed by pig and environment isolates in core SNP tree (Fig. 3) are also clustered together in this tree, which are labeled in text in the figure.

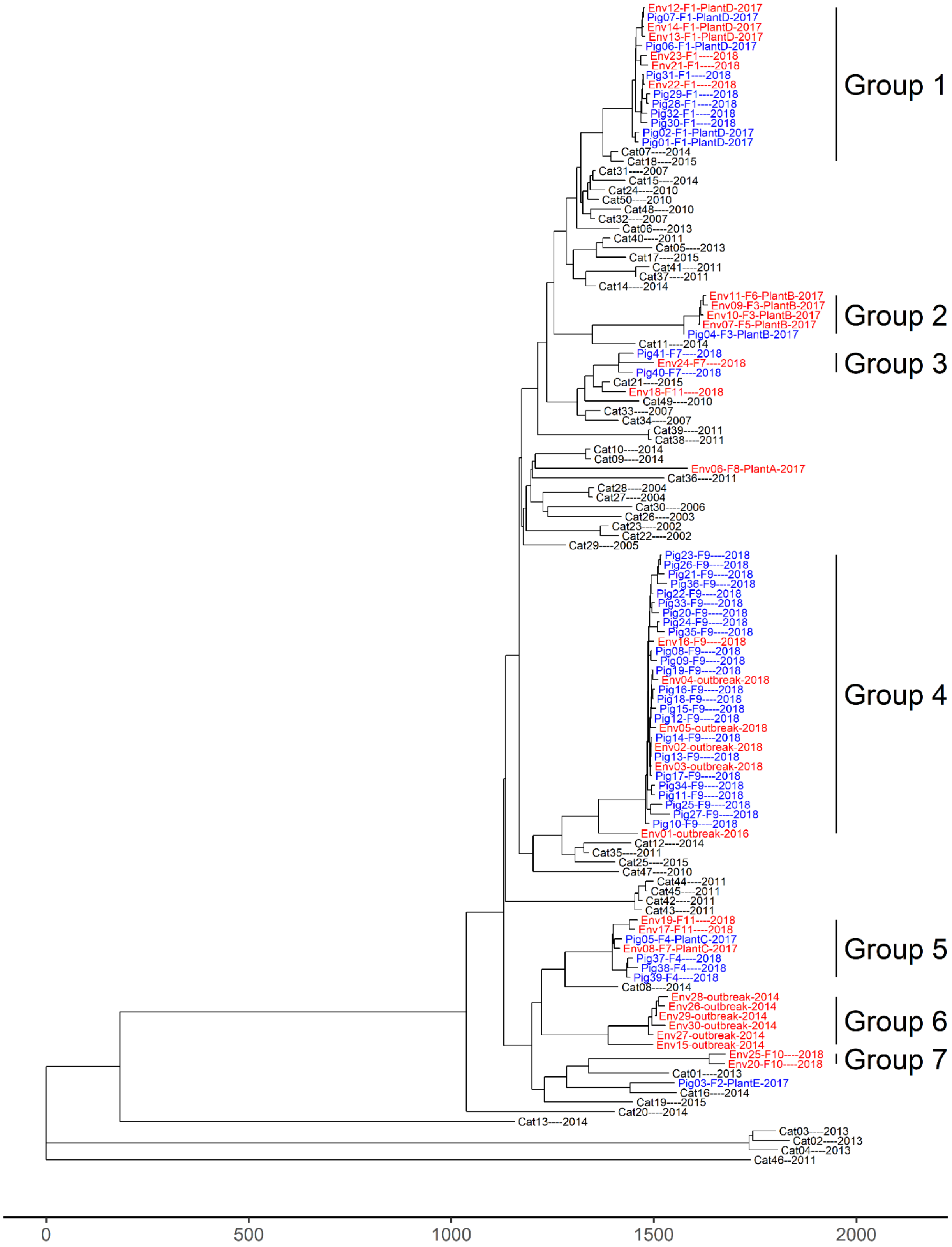
Env12-F1-PlantD-2017
Pig07-F1-PlantD-2017
Env14-F1-PlantD-2017
Env13-F1-PlantD-2017
Pig06-F1-PlantD-2017
Env23-F1----2018
Env21-F1----2018
Pig31-F1----2018
Env22-F1----2018
Pig29-F1----2018
Pig28-F1----2018
Pig32-F1----2018
Pig30-F1----2018
Pig02-F1-PlantD-2017
Pig01-F1-PlantD-2017
Cat07----2014
Cat18----2015
Cat31----2007
Cat15----2014
Cat24----2010
Cat50----2010
Cat48----2010
Cat32----2007
Cat06----2013
Cat40----2011
Cat05----2013
Cat17----2015
Cat41----2011
Cat37----2011
Cat14----2014
Env11-F6-PlantB-2017
Env09-F3-PlantB-2017
Env10-F3-PlantB-2017
Env07-F5-PlantB-2017
Pig04-F3-PlantB-2017
Cat11----2014
Pig41-F7----2018
Env24-F7----2018
Pig40-F7----2018
Cat21----2015
Env18-F11----2018
Cat49----2010
Cat33----2007
Cat34----2007
Cat39----2011
Cat38----2011
Cat10----2014
Cat09----2014
Env06-F8-PlantA-2017
Cat36----2011
Cat28----2004
Cat27----2004
Cat30----2006
Cat26----2003
Cat23----2002
Cat22----2002
Cat29----2005
Pig23-F9----2018
Pig26-F9----2018
Pig21-F9----2018
Pig36-F9----2018
Pig22-F9----2018
Pig33-F9----2018
Pig20-F9----2018
Pig24-F9----2018
Pig35-F9----2018
Env16-F9----2018
Pig08-F9----2018
Pig09-F9----2018
Pig19-F9----2018
Env04-outbreak-2018
Pig16-F9----2018
Pig18-F9----2018
Pig15-F9----2018
Pig12-F9----2018
Env05-outbreak-2018
Pig14-F9----2018
Env02-outbreak-2018
Pig13-F9----2018
Env03-outbreak-2018
Pig17-F9----2018
Pig34-F9----2018
Pig11-F9----2018
Pig25-F9----2018
Pig27-F9----2018
Pig10-F9----2018
Env01-outbreak-2016
Cat12----2014
Cat35----2011
Cat25----2015
Cat47----2010
Cat44----2011
Cat45----2011
Cat42----2011
Cat43----2011
Env19-F11----2018
Env17-F11----2018
Pig05-F4-PlantC-2017
Env08-F7-PlantC-2017
Pig37-F4----2018
Pig38-F4----2018
Pig39-F4----2018
Cat08----2014
Env28-outbreak-2014
Env26-outbreak-2014
Env29-outbreak-2014
Env30-outbreak-2014
Env27-outbreak-2014
Env15-outbreak-2014
Env25-F10----2018
Env20-F10----2018
Cat01----2013
Pig03-F2-PlantE-2017
Cat16----2014
Cat19----2015
Cat20----2014
Cat13----2014
Cat03----2013
Cat02----2013
Cat04----2013
Cat46--2011
Group 1
Group 2
Group 3
Group 4
Group 5
Group 6
Group 7
0
500
1000
1500
2000

11

**Figure S2.** A neighbor joining tree based on wgMLST profiles of 121 *E. coli* O157:H7 isolates. The wgMLST scheme included 24953 genes. The scale bar represents the allelic distance. Each tip in the tree was labeled with the isolate identification and if applicable, its associated farm (F), and/or processing plant (Plant) followed by the year of collection. The isolates recovered from pig (blue) and cattle (black) fecal samples and pig production environment samples (red) were distinguished using different colors. The seven groups formed by pig and environment isolates in core SNP tree (Fig. 3) are also clustered together in this tree, which are labeled in text in the figure.

A

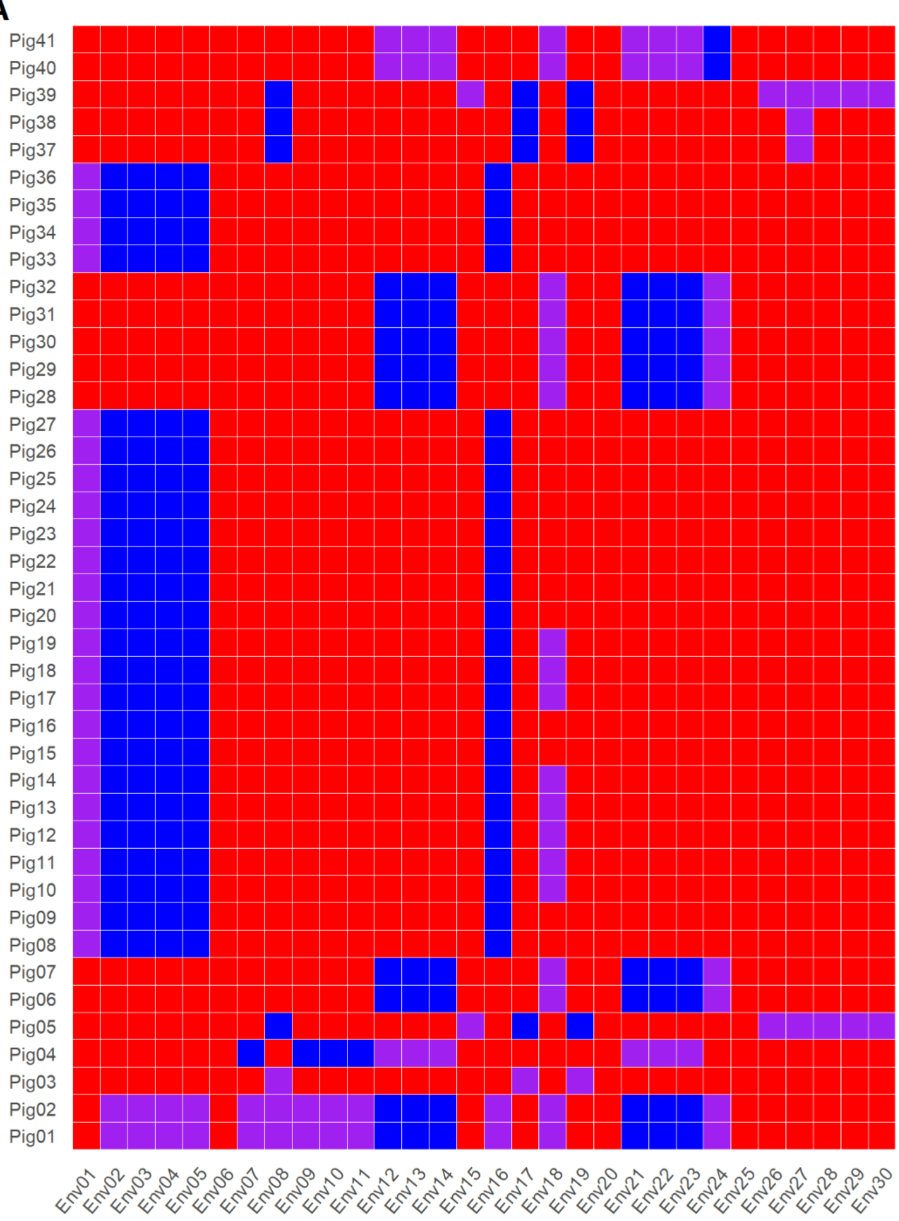
Pig41
Pig40
Pig39
Pig38
Pig37
Pig36
Pig35
Pig34
Pig33
Pig32
Pig31
Pig30
Pig29
Pig28
Pig27
Pig26
Pig25
Pig24
Pig23
Pig22
Pig21
Pig20
Pig19
Pig18
Pig17
Pig16
Pig15
Pig14
Pig13
Pig12
Pig11
Pig10
Pig09
Pig08
Pig07
Pig06
Pig05
Pig04
Pig03
Pig02
Pig01
Env01 Env02 Env03 Env04 Env05 Env06 Env07 Env08 Env09 Env10 Env11 Env12 Env13 Env14 Env15 Env16 Env17 Env18 Env19 Env20 Env21 Env22 Env23 Env24 Env25 Env26 Env27 Env28 Env29 Env30

19

B

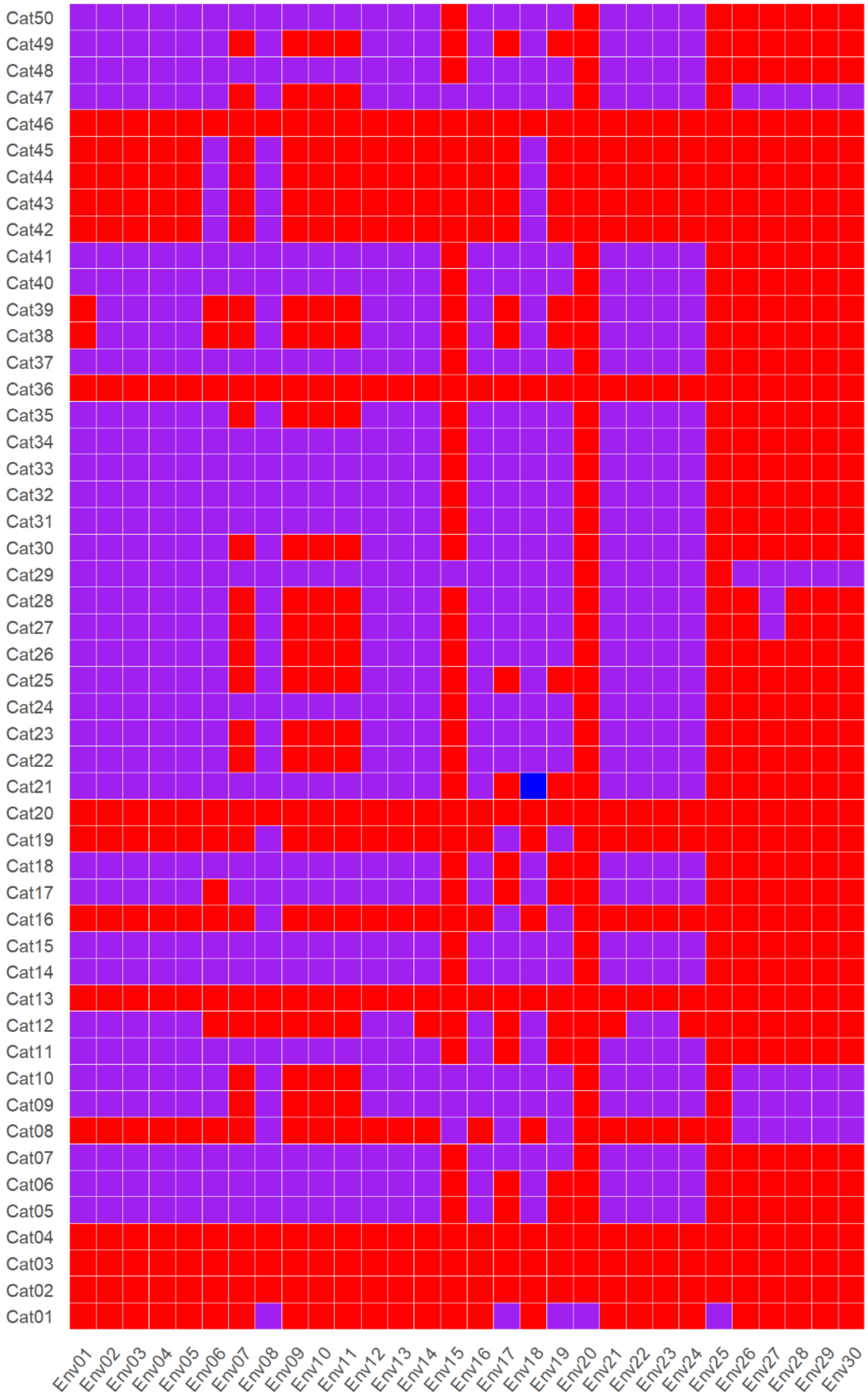
Cat50
Cat49
Cat48
Cat47
Cat46
Cat45
Cat44
Cat43
Cat42
Cat41
Cat40
Cat39
Cat38
Cat37
Cat36
Cat35
Cat34
Cat33
Cat32
Cat31
Cat30
Cat29
Cat28
Cat27
Cat26
Cat25
Cat24
Cat23
Cat22
Cat21
Cat20
Cat19
Cat18
Cat17
Cat16
Cat15
Cat14
Cat13
Cat12
Cat11
Cat10
Cat09
Cat08
Cat07
Cat06
Cat05
Cat04
Cat03
Cat02
Cat01
Env01
Env02
Env03
Env04
Env05
Env06
Env07
Env08
Env09
Env10
Env11
Env12
Env13
Env14
Env15
Env16
Env17
Env18
Env19
Env20
Env21
Env22
Env23
Env24
Env25
Env26
Env27
Env28
Env29
Env30

20

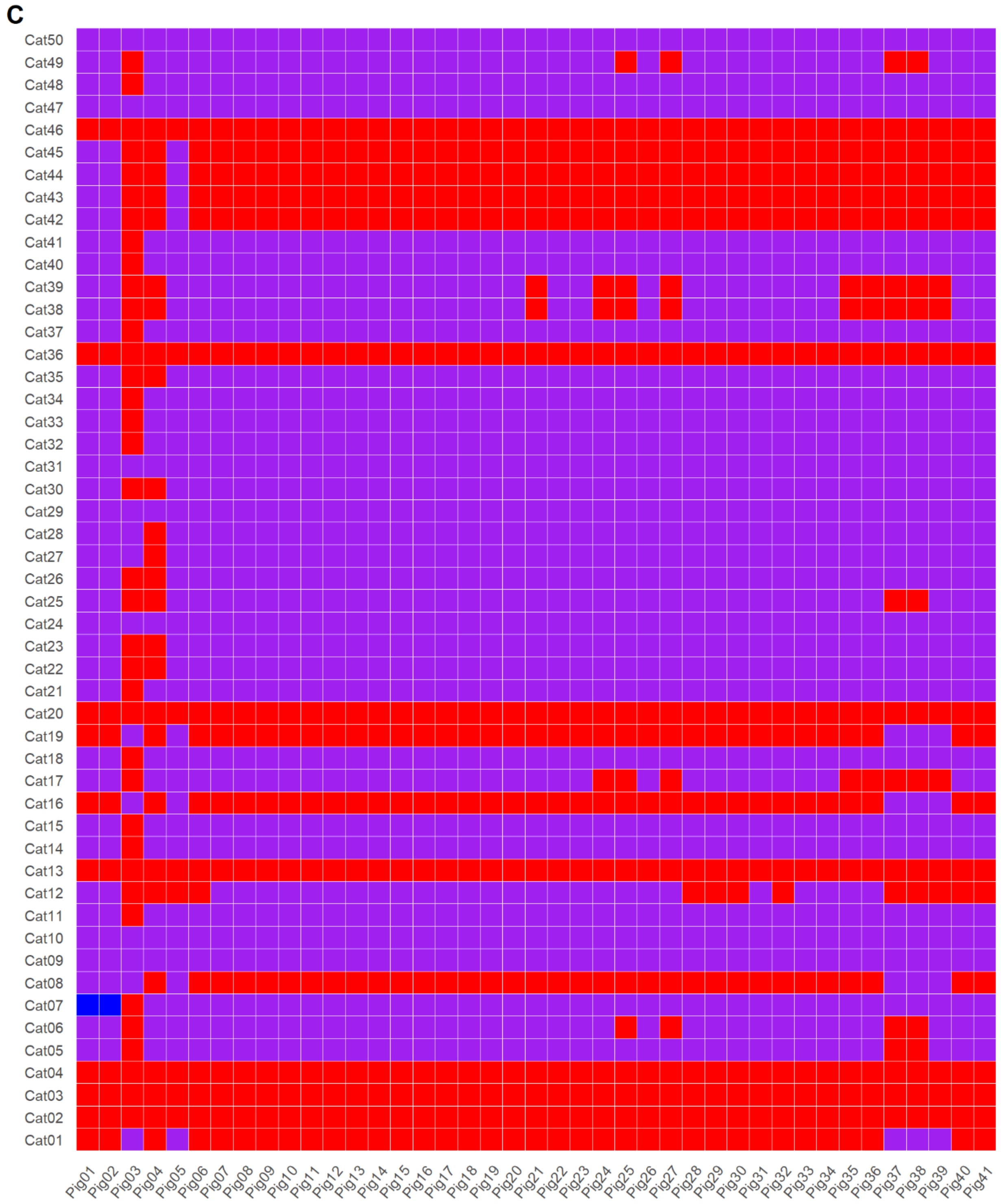


**Figure S3.** The SNP distance between pig and environmental isolates (Panel A), between cattle and environmental isolates (B) and between cattle and pig isolates (Panel C). The SNP distances

are shown with blue, purple and red representing < 21 SNPs, ≥ 21 SNPs and ≤ 100 SNP and > 100 SNPs, respectively.

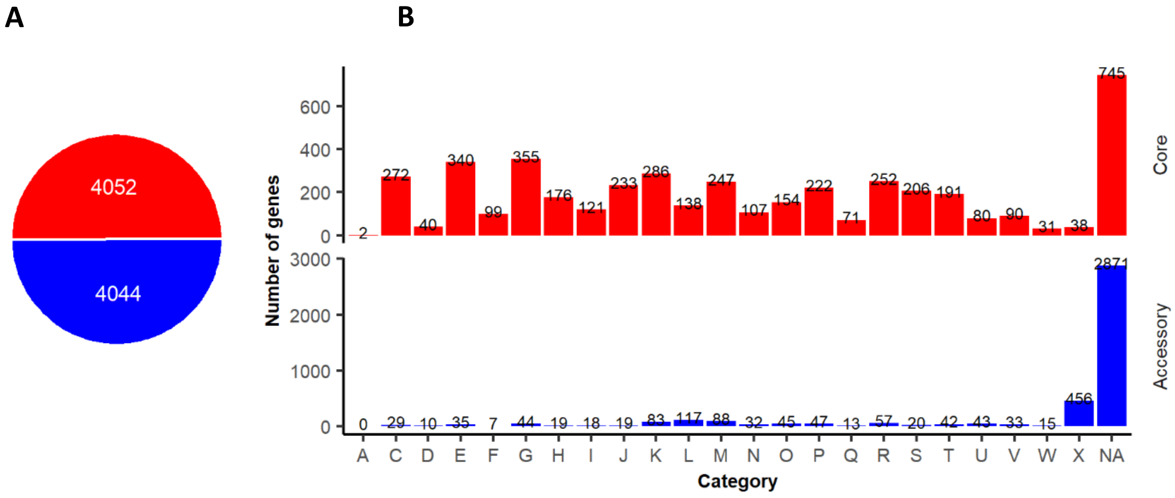


**Figure S4.** The function category of genes in the pan genome of O157:H7 isolates in this study. Panel A, the total number of core (red) and accessory (blue) genes. Panel B, the number of genes in each category in core and accessory genome. Each letter on the x-axis represent one functional category: A, RNA processing and modification; C, energy production and conversion, D, cell cycle control and mitosis; E, amino acid metabolism and transport; F, nucleotide metabolism and transport; G, carbohydrate metabolism and transport; H, coenzyme metabolism; I, lipid metabolism; J, translation; K, transcription; L, replication and repair; M, cell wall/membrane/envelop biogenesis; N, cell motility; O, post-translational modification, protein turnover, chaperone functions; P, inorganic ion transport and metabolism; Q, secondary structure; R, general functional prediction only; S, function unknown; T, signal transduction; U, intracellular trafficking and secretion; V, defense mechanisms; W, extracellular structures; X, mobilome: prophages, transposons. Of note, a number of genes were assigned into more than one categories

and hence counted more than once in the figure.
